# Risk factors for TB in Australia and their association with delayed treatment completion

**DOI:** 10.5588/ijtld.21.0111

**Published:** 2022-05-01

**Authors:** N. J. Coorey, L. Kensitt, J. Davies, E. Keller, M. Sheel, K. Chani, S. Barry, R. Boyd, J. Denholm, K. Watts, G. Fox, C. Lowbridge, R. Perera, J. Waring, B. Marais, K. Viney

**Affiliations:** 1 Australian National University Medical School, Canberra ACT, Australia; 2 Research School of Population Health, Australian National University College of Health and Medicine, Australian National University, Canberra ACT, Australia; 3 School of Public Health, The University of Sydney, Sydney, NSW, Australia; 4 Department of Global Public Health Sciences, Karolinska Institutet, Stockholm, Sweden; 5 South Australia Health, Adelaide, SA, Australia; 6 Northern Territory Health, Darwin, NT, Australia; 7 Victorian Tuberculosis Program, Melbourne Health, VIC, Australia; 8 Department of Infectious Diseases, Doherty Institute, The University of Melbourne, VIC, Australia; 9 Sydney Medical School-Central, The University of Sydney, Sydney, NSW, Australia; 10 Global and Tropical Health, Menzies School of Health Research, Charles Darwin University, Darwin, NT, Australia; 11 Centre for Research Excellence in Tuberculosis (TB-CRE), The University of Sydney, Sydney, NSW, Australia; 12 Marie Bashir Institute for Infectious Diseases and Biosecurity (MBI), The University of Sydney, Sydney, NSW, Australia; 13 Western Australia Health, Perth, WA, Australia; 14 Western Australia Tuberculosis Control Program, Perth, WA, Australia

**Keywords:** tuberculosis, TB, risk factors, surveillance, Australia

## Abstract

**BACKGROUND:**

Australia has a low incidence of TB and has committed to eliminating the disease. Identification of risk factors associated with TB is critical to achieving this goal.

**METHODS:**

We undertook a prospective cohort study involving persons receiving TB treatment in four Australian jurisdictions. Risk factors and their association with delayed treatment completion (treatment delayed by at least 1 month) were analysed using univariate analyses and multivariate logistic regression.

**RESULTS:**

Baseline surveys were completed for 402 persons with TB. Most (86.1%) were born overseas. Exposure to a person with TB was reported by 19.4%. Diabetes mellitus (10.2%), homelessness (9.2%), cigarette smoking (8.7%), excess alcohol consumption (6.0%) and mental illness (6.2%) were other common risk factors. At follow-up, 24.8% of patients had delayed treatment completion, which was associated with adverse events (34.1%, aOR 6.67, 95% CI 3.36–13.27), excess alcohol consumption (6.0%, aOR 21.94, 95% CI 6.03–79.85) and HIV co-infection (2.7%, aOR 8.10, 95% CI 1.16–56.60).

**CONCLUSIONS:**

We identified risk factors for TB and their association with delayed treatment completion, not all of which are routinely collected for surveillance purposes. Recognition of these risk factors should facilitate patient-centred care and assist Australia in reaching TB elimination.

## Abstract (fr)

**CONTEXTE:**

L’incidence de la TB en Australie est faible et le pays s’est engagé à éliminer la maladie. Afin d’atteindre cet objectif, il est essentiel d’identifier les facteurs de risque associés à la TB.

**MÉTHODES:**

Nous avons réalisé une étude de cohorte prospective auprès de personnes sous traitement antituberculeux dans quatre juridictions du pays. Les facteurs de risque et leur association avec un retard d’achèvement du traitement (d’au moins 1 mois) ont été analysés par analyses univariées et régression logistique multivariée.

**RÉSULTATS:**

Au total, 402 personnes ont répondu aux enquêtes initiales. La plupart (86,1%) étaient nées à l’étranger. Un contact avec un cas de TB a été rapporté par 19,4% d’entre elles. Diabète sucré (10,2%), absence de domicile fixe (9,2%), tabagisme (8,7%), consommation excessive d’alcool (6,0%) et troubles mentaux (6,2%) étaient d’autres facteurs de risque courants. Pendant la période de suivi, 24,8% des patients ont connu un retard d’achèvement du traitement, associé à des évènements indésirables (34,1%, OR ajusté [ORa] 6,67 ; IC 95% 3,36–13,27), une consommation excessive d’alcool (6,0% ; aOR 21,94 ; IC 95% 6,03–79,85) et à une co-infection par le VIH (2,7% ; aOR 8,10 ; IC 95% 1,16–56,60).

**CONCLUSIONS:**

Nous avons identifié les facteurs de risque de TB et leur association avec un retard d’achèvement du traitement, tous n’étant pas recueillis en routine à des fins de surveillance. L’identification de ces facteurs de risque devrait faciliter les soins centrés sur le patient et aider l’Australie à éliminer la TB.

TB continues to pose a major global public health challenge. Although the vast majority of this disease burden occurs in low- and middle-income countries,^1^,^2^ high-income countries also grapple with optimal case management of TB, especially in high-risk populations. TB is associated with multiple social determinants.^3^,^4^ Careful consideration of these determinants may provide insights that can assist in optimisation of public health strategies and patient-centred approaches.^5^,^6^

Australia reports one of the lowest incidence rates of TB globally (5.8 cases per 100,000 population in 2018).^7^ Rates have been static since the mid-1980s. Annually, approximately 1,400 TB cases are notified, with nearly 90% of cases identified in persons born overseas.^8^ Australia’s migration intake includes people from high TB incidence countries (defined as >40 cases/100,000).^1^,^2^ While most people are screened for active TB before arriving in Australia, some have undiagnosed TB infection on arrival. Also, many frequently return to their country of origin, which provides an ongoing source of exposure and infection.^9^ Australia has published its strategic plan to control TB,^8^ which aims to achieve TB elimination (defined as <1 case/million) by the year 2050.^1^,^2^,^8^

TB is a notifiable disease in Australia.^10^ Jurisdictions (states and territories) report these data according to a pre-defined protocol to the National Notifiable Diseases Surveillance System (NNDSS).^11^ Approximately 15 variables are collected for TB in the NNDSS, including HIV status, household TB contact, health industry employment within the past 5 years, and past residence (≥3 months) in a high TB incidence country.^12^ Some other important variables are not recorded in the NNDSS, but may be accessible through medical case notes or other information systems which, if analysed, may assist policy makers and those working in TB programmes to refine efforts to provide patient-centred care and eliminate TB.

In this study, we collected information on risk factors associated with TB disease and treatment outcomes in a sample of patients treated for TB in Australia. This included risk factors not routinely captured in the NNDSS. Our purpose was to inform the delivery of patient-centred care in Australia and other low TB incidence settings.

## METHODS

### Study design

We undertook a prospective cohort study of all persons with TB who received treatment for TB on 1 November 2018 in Victoria (VIC), South Australia (SA) and the Northern Territory (NT), and on 1 August 2019 in Western Australia (WA). This study included a baseline questionnaire with a follow-up survey after 12 months.

### Study population

All persons with confirmed TB from four Australian jurisdictions (VIC, SA, NT and WA) were included. Jurisdictional TB programme managers compiled a list of persons with TB who were receiving treatment, or who were due to receive treatment on a specified day which provided a cross-sectional sample of all persons undergoing TB treatment.

In Australia, all patients notified to a jurisdictional TB surveillance system fulfil national TB case definition criteria as specified by the Australian Commonwealth Department of Health.^13^ These case definition criteria require a diagnosis accepted by the Director of Tuberculosis Control (or equivalent) in the relevant jurisdiction, based on either 1) definitive laboratory evidence, or 2) clinical evidence. Definitive laboratory evidence is defined as 1) isolation of *Mycobacterium tuberculosis* complex (*M. tuberculosis, M. bovis* or *M. africanum*, excluding *M. bovis* var bacille Calmette-Guérin) using culture, or 2) detection of *M. tuberculosis* complex using nucleic acid testing, except where this is likely to be due to previously treated or inactive disease.^13^ Clinical evidence is defined as a clinical diagnosis of TB, including clinical follow-up assessment to ensure a consistent clinical course, by a clinician experienced in TB management.

### Data collection

Two paper-based questionnaires (Supplementary Data 1.1, 1.2) were developed to collect information on risk factors associated with TB at baseline, as well as treatment outcomes 12 months later. The risk factor variables were based on a review of current risk factor variables used for TB surveillance in Australia, as well as a comprehensive literature search identifying additional risk factors not routinely captured in the NNDSS.

Variables in the baseline questionnaire included risk factors such as homelessness, history of incarceration, cigarette smoking, diabetes mellitus, HIV status, illicit drug use and excessive alcohol use, as well as routinely collected data such as age, sex and country of birth.^14^ The follow-up questionnaire collected information on TB treatment outcomes, including cure, completion, failure and death, for which NNDSS definitions were applied (i.e., dataset field specifications v6.2.1; personal communication, Data Manager, NNDSS, January 2020). Both questionnaires were piloted by TB case managers in VIC using information from a small group of persons with TB who had completed treatment.

Baseline and follow-up data (12 months from baseline) were collected in VIC, SA and NT. Collection of data in other Australian jurisdictions and follow-up data in WA were not completed due to the time required for ethics approvals; the different dates for the baseline questionnaires reflect the timing of ethics approvals. Questionnaires were completed by trained data collectors, including TB nurses or physicians responsible for patient management. Questionnaires were completed based on information in medical records and within jurisdictional TB surveillance systems. Following completion of the questionnaires, data were checked by senior TB programme staff and were sent via a secure route to study investigators. Data were entered into a restricted MS Access database (MicroSoft, Redmond, WA, USA) on a password protected server, with in-built data quality checks. A unique identifier was allocated to each patient. Any missing, unclear or inconsistent data were checked with jurisdictional TB programme staff and were corrected.

### Data analysis

We conducted descriptive analyses using numbers and proportions. Statistical comparison between the study sample and the 2018 Australian population with TB was conducted using Pearson’s χ^2^ test and Fisher’s exact test, in case of small numbers. We conducted univariate analyses, calculating odds ratios (ORs) and 95% confidence intervals (95% CIs) for the association between risk factors and delayed treatment completion. This was defined as treatment that was not completed within 1 month of the planned treatment completion date for a given regimen (e.g., drug-susceptible or drug-resistant TB). Drug resistance categories in our analyses were classified according to WHO guidelines.^15^

Selected variables (*P* < 0.4) were included in a multivariate regression model and controlled for the effects of age and sex, with backwards elimination at a 0.05 significance level. This model was used to calculate adjusted ORs (aORs). All data were analysed using Stata Statistical Software: Release 15.1 (StataCorp, College Station, TX, USA; 2017).

### Ethical issues

A summary of all ethical and site-specific approvals obtained is provided in Supplementary Data 1.3. A waiver of consent was sought and approved as the research involved analysis of data gathered under Public Health Acts within local jurisdictions for the purpose of legislated activities under these Acts, rather than for research purposes.

## RESULTS

### Demographic characteristics

Baseline data were available for 402 persons with TB from VIC (*n* = 277, 68.9%), WA (*n* = 66, 16.4%), SA = 42, 10.4%) and the NT (*n* = 17, 4.2%). Follow-up data at 12 months were available for 331 persons with TB (82.3%), excluding patients from WA (see Methods).

Demographic and clinical characteristics of the 402 persons with TB are given in Tables 1 and 2. The demographic characteristics of the sample were not significantly different from the overall Australian population with TB for 2018 (Table 1). Our study sample included 201 females (50.0%), with a median age of 35 years. Most (*n* = 346, 86.1%) were born overseas and 6 (1.5%) were of Aboriginal and/or Torres Strait Islander (Indigenous) origin. One quarter (*n* = 101, 25.1%) were not eligible for the Medicare Benefits Scheme (MBS), whereby the Australian Government pays a rebate to subsidise the cost of medical services (Table 2). Those not eligible for the MBS were primarily overseas students (54.5%), visitors (18.8%) and those with other types of VISAs (13.9%). Thirty-one (7.7%) patients had drug-resistant TB, including five with multidrug-resistant TB (MDR-TB; defined as TB resistant to at least rifampicin and isoniazid) and five with extensively drug-resistant TB (XDR-TB; defined as MDR-TB with additional resistance to a fluoroquinolone and at least one of the second-line injectable agents [amikacin, kanamycin, or capreomycin]). Additional information describing immigration status and travel history is provided in Supplementary Data 1.4.

**Table 1:** Sociodemographic characteristics of all patients with TB in Australia (2018) compared to 402 patients from Victoria, Western Australia, South Australia and the Northern Territory (2018–2019)

Patient characteristics	Study sample (*n* = 402) *n* (%)	All TB patients^*^ (*n* = 1438)	

		*n* (%)	*P* value
Age group, years			0.006^†^
0–14	28 (7.0)	49 (3.4)	
15–34	171 (42.5)	606 (42.1)	
35–64	148 (36.8)	544 (37.8)	
≥65	54 (13.4)	239 (16.6)	
Unknown	1 (0.2)	0 (0)	
Sex (2014 data)			0.344
Female	201 (50.0)	627 (46.8)	
Male	200 (49.8)	711 (53.1)	
Other	1 (0.2)	1 (0.1)	
Indigenous status^‡^			0.507
Non-indigenous	393 (97.8)	1409 (98.0)	
Indigenous	6 (1.5)	29 (2.0)	
State or Territory of notification			0.201
Victoria	277 (68.9)	450 (65.5)	
Western Australia	66 (16.4)	136 (19.8)	
South Australia	42 (10.4)	83 (12.1)	
Northern Territory	17 (4.2)	18 (2.6)	
Country of birth			0.326
Born overseas	346 (86.1)	1276 (88.7)	
Born in Australia	56 (13.9)	161 (11.2)	
Unknown	0 (0.0)	1 (0.1)	

^*^ All patients reported to the National Notifiable Diseases Surveillance System.

^†^Statistically significant.

^‡^Persons of Aboriginal and Torres Strait Islander descent (*n* = 399; 3 with missing data).

**Table 2:** Social and clinical characteristics of surveyed Australian TB patients

Characteristics	(*n* = 402) *n* (%)
Demographic characteristics	
Employment status	
Unemployed	216 (53.7)
Full time employed	110 (27.4)
Part time employed	40 (10.0)
Casual	27 (6.7)
Unknown	9 (2.2)
Language spoken during clinic visits^*^	
English	326 (81.1)
Language other than English	70 (17.4)
Unknown	6 (1.5)
Eligibility for medical benefits scheme	
Eligible	293 (72.9)
Non-eligible	101 (25.1)
Unknown	8 (2.0)
Household size (number of persons, including the patient)	
1–5	294 (73.1)
≥6	100 (24.9)
Unknown	8 (2.0)
Clinical characteristics	
TB site	
Pulmonary only	191 (47.5)
Extrapulmonary only	146 (36.3)
Both	64 (15.9)
Unknown	1 (0.2)
Diagnostic tests used to confirm diagnosis^†^	
Culture	280 (69.7)
Molecular test	263 (65.4)
Sputum smear microscopy	158 (39.3)
Whole-genome sequencing	127 (31.6)
Histology	104 (25.9)
Microscopy on non-sputum sample	59 (14.7)
Diagnosis made on clinical assessment only	36 (9.0)
Drug resistance status (*n* = 294^‡^)	
Drug-susceptible^§^	263 (89.5)
Mono-resistant^¶^	21 (7.1)
MDR/RR-TB	5 (1.7)
XDR-TB	5 (1.7)
Prior history of TB treatment	
No prior history of TB treatment	392 (97.5)
Prior history of TB treatment^#^	9 (2.2)
Unknown	1 (0.2)

^*^ This variable was collected as a proxy for the language used most frequently by the patient.

^†^Multiple diagnostic methods could be used in one patient.

^‡^*N*= 294 as drug resistance status was not available for 108 patients.

^§^Drug susceptibility defined as susceptibility to first-line TB medicines; determination attempted in all culture-confirmed cases.

^¶^Defined as resistance to one of the first-line TB medicines (i.e., rifampicin, isoniazid, pyrazinamide or ethambutol). All 21 patients with mono-resistance were resistant to isoniazid. There were no cases of rifampicin mono-resistance.

^#^Four patients received full treatment in Australia, four received partial treatment overseas and one received full or partial treatment overseas. MDR = multidrug-resistant; RR-TB = rifampicin-resistant TB; XDR-TB = extensively drug-resistant TB.

### Risk factors

Data on risk factors are presented in Table 3. Known contact with a person with TB was the most common risk factor (*n* = 78, 19.4%), followed by employment in the health or aged care sector (*n* =37, 9.2% overall and 20.7% of the employed sample of 177 persons). Of the 402 people with TB, 37 (9.2%) reported homelessness (currently or in the previous 2 years); 24 (6.0%) were reported to drink alcohol excessively; 35 (8.7%) were current smokers and 58 (14.4%) reported having smoked previously; 25 (6.2%) reported having a mental illness; 19 (4.7%) reported illicit drug use (three of these reported intravenous drug use) and 5 (1.2%) had a history of previous incarceration.

**Table 3:** Reported risk factors of surveyed Australian TB patients

Risk factors for TB	(*n* = 402) *n* (%)
Employed (*n* = 177)	
Employment in healthcare	25 (14.1)
Employment in aged care	12 (6.8)
Previous healthcare employment	9 (5.1)
Employment in childcare	1 (0.6)
Lives in aged care facility	
No	323 (80.3)
Yes	4 (1.0)
Unknown	75 (18.7)
Ever incarcerated	
No	363 (90.3)
Yes	5 (1.2)
Unknown	34 (8.5)
Homelessness (current or during last 2 years)	
No	347 (86.3)
Yes	37 (9.2)
Unknown	18 (4.5)
Known contact with a TB patient	
No	303 (75.4)
Yes	78 (19.4)
Unknown	21 (5.2)
Smoking status	
Never smoker	231 (57.5)
Ever smoker	58 (14.4)
Current smoker	35 (8.7)
Unknown	78 (19.4)
Illicit drug use	
No	352 (87.6)
Yes^*^	19 (4.7)
Unknown	31 (7.7)
Excess alcohol consumption	
No	341 (84.8)
Yes	24 (6.0)
Unknown	37 (9.2)
Diabetes mellitus	
No	354 (88.1)
Yes	41 (10.2)
Unknown	7 (1.7)
HIV status	
HIV-negative	329 (81.8)
HIV-positive	11 (2.7)
Unknown	62 (15.4)
On immunosuppressive treatment prior to TB treatment	
No	330 (82.1)
Yes	26 (6.5)
Unknown	46 (11.4)
Mental illness	
No	351 (87.3)
Yes	25 (6.2)
Unknown	26 (6.5)
Chronic renal disease	8 (2.0)

^*^ Includes three patients reporting intravenous drug use and 16 patients reporting non-intravenous drug use.

The HIV status of most (84.5%) patients was known, but unknown for 15.5%; 11 patients were HIV-positive (2.7%). Overall, 8 had hepatitis C (2.0%), 5 had hepatitis B (1.2%) and 3 had both hepatitis B and C virus infection (0.7%). Four patients had both HIV and concomitant hepatitis B or C infection. In addition, 41 (10.2%) had diabetes mellitus, 26 (6.5%) reported use of an immunosuppressant medication and 8 had chronic kidney disease (2.0%). Five women (2.5% of all women) were pregnant at the time of TB diagnosis. Additional information on TB treatment and care, including health service utilisation, is provided in Supplementary Data 1.4.

### Treatment outcomes

Follow-up data on treatment outcomes were collected for 331 patients from VIC, SA and the NT (Table 4). Most patients completed treatment (*n* =294, 88.8%), with 214 (64.7%) of patients completing treatment within 1 month of the planned completion date. One patient died from heart failure 7 months after TB treatment started. Forty-one patients (12.4%) reported economic or social consequences related to TB. Feeling socially isolated (3.9%) and perceived stigma (2.7%) were noted, but were uncommon.

**Table 4:** Treatment outcomes of surveyed Australian TB patients

Treatment characteristics	(*n* = 331)^*^ *n* (%)
Treatment outcome^†‡^	
Completed	267 (80.7)
Cured	27 (8.2)
Ongoing	27 (8.2)
Transferred overseas	8 (2.4)
Loss to follow-up	1 (0.3)
Death^§^	1 (0.3)
Treatment completion delayed by at least 1 month^¶^	
No	214 (64.7)
Yes	82 (24.8)
Unknown	35 (10.6)
Reported adverse economic or social consequences of treatment	
No	290 (87.6)
Yes^#^	41 (12.4)
Job/working hours lost	16 (4.8)
Social isolation	13 (3.9)
Perceived stigmatisation	9 (2.7)
Interrupted studies	7 (2.1)
Difficulties affording services	4 (1.2)
Other	6 (1.8)

^*^ At the time of the follow-up survey, these patients were still receiving TB treatment.

^†^ Treatment outcome data from Western Australia were not collected for this study.

^‡^ Other treatment outcomes are possible (i.e., treatment failed, not followed up, outcome unknown). However, there were no patients with these outcomes reported.

^§^ Patient passed away from heart failure 7 months after commencing treatment for TB.

^¶^ Defined as treatment delayed by at least 1 month past the planned treatment completion date. The planned treatment completion date may vary according to the TB resistance status and pattern.

^#^ Multiple social and economic consequences may occur in a single patient.

### Delayed tuberculosis treatment completion

There were 82 patients (24.8%) for whom treatment completion was delayed by at least 1 month. Table 5 shows the association between selected characteristics and risk factors with delayed treatment completion. Delayed treatment completion was reported for patients with excessive alcohol intake (79.0%), illicit drug users (73.3%), persons living with HIV (70.0%), Indigenous patients (60.0%), patients who had experienced homelessness in the past 2 years (56.7%) and those with mental illness (58.8%). Furthermore, treatment completion was delayed in over 50% of patients with drug-resistant TB, including those with mono-resistant (52.9%), as well as MDR- or XDR-TB (66.7%). In our multivariable regression, adverse events during treatment (aOR 6.67, 95% CI 3.36–13.27; *P* < 0.001), excessive alcohol consumption (aOR 21.94, 95% CI 6.03–79.85; *P* < 0.001) and HIV co-infection (aOR 8.10, 95% CI 1.16–56.60; *P* = 0.05) were associated with delayed TB treatment completion.

**Table 5:** Association between patient and social characteristics and delayed TB treatment ^*^ outcome of surveyed Australian TB patients ^†^

Risk factors (*n* = 82 patients with a delayed TB treatment outcome)^‡^	*n/N* (%)^§^	OR (95% CI)	*P* value	aOR^¶^ (95% CI)	*P* value
Age group, years (*n* = 295)					
0–14	7/23 (30.4)	1.23 (0.46–3.32)	0.68	2.83 (0.70–11.38)	0.14
15–34	38/133 (28.6)	1.12 (0.63–2.01)	0.69	1.70 (0.79–3.69)	0.17
35–64	27/103 (26.2)	Reference		Reference	
≥65	10/36 (27.8)	1.08 (0.46–2.54)	0.86	0.99 (0.31–3.20)	0.99
Sex (*n* = 295)					
Female	39/146 (26.7)	Reference		Reference	
Male	43/149 (28.9)	1.11 (0.67–1.85)	0.68	1.06 (0.54–2.08)	0.87
Country of birth					
Australian-born	14/45 (31.1)	Reference			
Overseas-born	68/251 (27.1)	0.82 (0.41–1.64)	0.58		
Any healthcare employment	4/20 (20.0)	0.63 (0.21–1.96)	0.43		
Drug resistance (*n* = 203)					
Drug-susceptible	51/183 (27.9)	Reference			
Monoresistance	9/17 (52.9)	3.33 (1.22–9.09)	0.02^#^		
MDR/XDR-TB	2/3 (66.7)	5.92 (0.53–66.68)	0.15		
Supervision (*n* = 294)					
Directly observed treatment	33/106 (31.1)	Reference			
Self-administered therapy	47/188 (25.0)	0.74 (0.44–1.25)	0.26		
Adverse events documented during treatment	49/103 (47.6)	4.65 (2.70–8.03)	<0.001^#^	6.67 (3.36–13.27)	<0.001^#^
Adherence support measures during treatment^**^	66/248 (26.6)	1.53 (0.42–5.58)	0.52		
TB type (*n* = 295)					
Pulmonary TB	41/144 (28.5)	Reference			
Extrapulmonary TB	27/105 (25.7)	0.87 (0.49–1.53)	0.63		
Both	13/46 (28.3)	0.99 (0.47–2.07)	0.98		
High alcohol consumption	15/19 (79.0)	12.66 (4.04–39.67)	<0.001^#^	21.94 (6.03–79.85)	<0.001^#^
Any illicit drug use	11/15 (73.3)	9.50 (2.92–30.98)	<0.001^#^		
Homelessness, current or last 2 years	17/30 (56.7)	4.16 (1.91–9.06)	<0.001^#^		
Smoking status (*n* = 238)					
Never smoked	36/177 (20.3)	Reference			
Ex-smoker	15/39 (38.5)	2.45 (1.17–5.14)	0.02^#^		
Current smoker	12/22 (54.6)	4.70 (1.88–11.74)	0.001^#^		
Diabetes mellitus	12/28 (42.9)	2.10 (0.95–4.66)	0.07		
HIV status (*n* = 296)					
Negative	59/235 (25.1)	Reference		Reference	
Positive	7/10 (70.0)	6.96 (1.74–27.79)	0.006^#^	8.10 (1.16–56.60)	0.04^#^
Unknown	16/51 (31.4)	1.36 (0.70–2.64)	0.36	1.25 (0.48–3.21)	0.65
Mental illness (*n* = 17)	10/17 (58.8)	4.33 (1.58–11.85)	0.004^#^		

^*^ Delayed treatment completion was defined as treatment delayed by at least 1 month past the planned treatment completion date. The planned treatment completion date may vary according to the TB resistance status and pattern.

^†^*N* = 296 as patients with ongoing treatment or who transferred overseas were not identified as having an outcome relating to time of treatment completion. Eight patients were transferred overseas and 27 patients had ongoing treatment at the time of survey.

^‡^ Note that some risk factors included in Table 3 were omitted from this table due to low count numbers.

^§^ Proportion of patients with the risk factor who had delayed treatment completion, i.e., there were 10 HIV patients identified from the follow-up survey, and seven (70%) of these had delayed treatment completion. The number of persons with TB with delayed treatment completion overall was 82.

^¶^ Odds were adjusted for age group, sex, adverse events during treatment, high alcohol consumption and HIV status.

^#^ Statistically significant.

^**^ The adherence support measures included video directly observed treatment, SMS adherence support and other methods of adherence support. OR = odds ratio; CI = confidence interval; aOR = adjusted OR; MDR/XDR-TB = multidrug/extensively drug-resistant TB.

## DISCUSSION

TB is one of a small number of notifiable diseases in Australia for which enhanced surveillance is conducted. The NNDSS captures a specified number of data variables and risk factors for each person with TB, as recommended and endorsed by the National Tuberculosis Advisory Committee. This study identified multiple risk factors among persons with TB in Australia, not all of which are routinely collected in the NNDSS. TB treatment completion overall was high (89%), but a quarter of persons had delayed treatment completion. This delay was significantly associated with excessive alcohol use, HIV co-infection and adverse events during TB treatment. Some form of treatment support was provided to approximately three quarters (74.6%) of patients (Supplementary Data 3).

Excessive alcohol consumption may be a risk factor of under-appreciated significance. In our study, the majority (78.9%) of persons with TB with excessive alcohol consumption had delayed treatment completion (OR 12.43, 95% CI 3.97–38.96). Other studies have shown that alcohol misuse contributes to approximately 10% of TB deaths globally.^16^,^17^ Alcohol is a dose-dependent risk factor, with consumption of more than 40 g of alcohol per day (or a diagnosis of alcohol use disorder) resulting in a nearly three-fold increase in TB risk.^16^,^17^ Alcohol can negatively affect treatment adherence and may increase the risk of adverse events, particularly hepatotoxicity.^18^

In our study, persons with TB had a higher prevalence of HIV infection (2.7%) than the general Australian population (~0.1%).^19^ HIV testing and care remains important in low TB incidence settings, despite the fact that HIV-associated TB is substantially lower than the global population (8.2%). HIV is an important risk factor for developing TB, even in countries with low TB incidence.^1^,^20^–^22^ Most people with TB and HIV co-infection (70%) had delayed treatment completion (OR 6.84, 95% CI 1.72–27.31). Reasons for the delay remain unclear and warrant further investigation, but may have been influenced by a low threshold to extend treatment in immunocompromised persons. The current WHO recommendation is that a standard 6-month regimen is effective in treating people with HIV and drug-susceptible pulmonary TB, provided they are taking antiretroviral treatment.^23^–^28^ Only one patient with TB and HIV co-infection in our cohort had drug-resistant TB, which was mono-resistant to isoniazid. Other possible explanations for the delay include drug–drug interactions, problems adhering to treatment, unnecessary prolongation of treatment and difficulties accessing healthcare.^29^,^30^

Documented adverse events were also associated with delayed treatment completion. Such events are not uncommon during TB treatment, particularly when treating drug-resistant strains, as patients may be required to stop certain medications or adjust the treatment regimen.^28^ This potentially complicates and extends treatment.

A study that included all of Australia’s states and territories would have provided a more comprehensive analysis of TB risk factors in the Australian TB patient population. Although we collected data from a subset of jurisdictions, our study sample was similar to the most recent published data (2018) on the Australian TB population. We only found a minor difference in the age composition, with more people aged 0–14 years and fewer people aged ≥65 years in our sample. We were unable to perform sub-analysis by jurisdiction due to small sample sizes. Another limitation was that we did not stratify by place of birth, i.e., whether Australianborn (Indigenous or non-Indigenous) or overseas-born. Indigenous populations are disproportionately affected by TB globally, even in low TB incidence countries such as Australia, where the rate of TB in the Indigenous Australian-born population is four-fold higher than in non-Indigenous Australian-born people (but lower than in overseas-born persons).^31^,^32^ Owing to our relatively small sample size of Indigenous Australians (*n* = 6), we did not undertake a separate analysis for this group. A larger sample size would permit stratification.

This study has potential implications for TB management in Australia and other low TB incidence countries. Although Australia is a high-income country, 12.4% of patients reported economic or social consequences of treatment, which merits further attention. In addition, based on our study findings, we recommend the inclusion of the most important risk factors as part of routine TB data surveillance or the collection of these data during periodic surveys.^11^ This should include variables such as the presence/absence of diabetes mellitus, excessive alcohol use, cigarette smoking and substance use. Future studies could aim to investigate which risk factors coexist most commonly and their relationship to treatment outcomes. Furthermore, smoking cessation and support for people with excessive use of alcohol or illicit drug use should be considered during TB treatment,^33^,^34^ as well as psychological support.^28^,^35^ Collecting data about factors related to TB risk and patient-centred care should further improve patient outcomes (such as timely treatment completion and health-related quality of life), refine efforts to prevent TB among high-risk groups and focus TB elimination efforts in low incidence settings, such as Australia.
